# TEMPL: A Template-Based Protein–Ligand Pose Prediction Baseline

**DOI:** 10.1021/acs.jcim.5c01985

**Published:** 2025-10-14

**Authors:** Jozef Fülöp, Martin Šícho, Wim Dehaen

**Affiliations:** 1 CZ-OPENSCREEN, Department of Informatics and Chemistry, Faculty of Chemical Technology, 52735 University of Chemistry and Technology Prague, Technická 5, Prague 6 16 628, Czech Republic; 2 Department of Organic Chemistry, Faculty of Chemical Technology, 52735 University of Chemistry and Technology Prague, Technická 5, Prague 6 16 628, Czech Republic

## Abstract

Pose prediction of ligands to proteins remains a central challenge of structure-based drug design. Although data leakage and generalizability concerns remain, data-driven methods for pose prediction (i.e., based on deep learning and diffusion) now routinely outperform traditional techniques such as molecular docking. In this work, we propose a simple data-driven ligand-based baseline for pose prediction, which is based on maximal common substructure to reference molecules, followed by constrained 3D embedding. As this TEMplate-based Protein–Ligand (TEMPL) baseline is strictly data-driven, it is a particularly meaningful baseline for interpolative tasks, where physics-based methods sometimes underperform as they exploit data less directly. However, it can also highlight the added advantage of other interpolative data-driven methods that should outperform this simple approach. We applied our baseline method in the ASAP-Polaris-OpenADMET antiviral competition, achieving a result that outperformed some classic docking algorithms for the pose prediction of a series of ligands at the Main Protease of SARS-CoV-2 and MERS-CoV. Furthermore, we show that the performance of our baseline is relatively good on a protein–ligand pose prediction benchmark used for deep learning based pose prediction, PDBBind, highlighting the risk of data leakage and the necessity of challenging splits for other data-driven methods as well. We also show our baseline method has limited performance on more challenging benchmarks, such as PoseBusters. We provide our baseline method as open source software. For convenience and for nontechnical users, we also provide a web application to run the pipeline. These findings will aid in the evaluation of future pose prediction methods, especially more complex data-driven approaches that are increasing in popularity.

## Graphical abstract

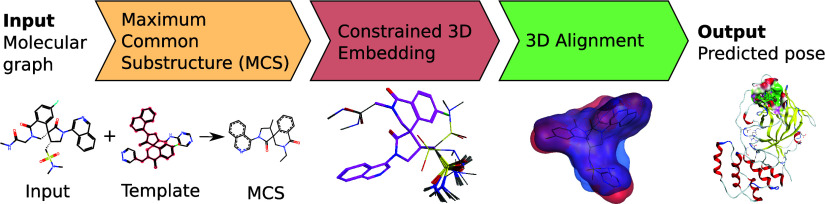

## Introduction

The prediction of the pose (conformation and absolute placement of a ligand in a protein binding site) is one of the grand challenges of structure-based drug design.^1^ The capacity to predict the correct pose of a ligand enables rational design of analogs, as well as providing the starting point for molecular modeling such as molecular dynamics or free energy of binding estimation methods. The traditional method used for pose prediction is molecular docking, which is widely used and has achieved many successes as an important component of drug design campaigns,^2^ but which also has several well-known serious limitations.^3,4^ Molecular docking is based on the combination of a scoring function, which ranks a given pose, and a search method, which is used to optimize starting poses into the lowest possible score, usually using a heuristic approach.^5^

Recently, several new data-driven methods for pose prediction that are distinct from classic molecular docking^6,7^ were developed, which can be broadly divided into two main categories: (1) deep learning based pose prediction and (2) cofolding. In deep learning based pose prediction, large protein–ligand complex structure data sets are leveraged to predict ligand poses using deep learning methods. Some notable methods are EquiBind,^8^ which is based on E(3)-equivariant geometric deep learning, and DiffDock,^9^ a diffusion based approach. Another early pose prediction method based on deep learning was TankBind, using trigonometry aware neural networks.^10^ Conversely, in cofolding, protein 3D structure prediction and ligand placement are done concurrently, meaning the input is the sequence of the target protein and not a pre-existing 3D structure. The best known example of the cofolding approach is AlphaFold3 ^11^ as well as closely related approaches such as Chai,^12^ Boltz,^13^ Protenix,^14^ NeuralPlexer^15^ as well as RosettaFold AllAtom.^16^ These methods have all achieved superior performance in pose prediction success on common benchmarks.

A typical problem of data-driven machine learning approaches is limited extrapolative capacity. Analysis showed that the good reported performance of deep learning based pose prediction and cofolding can be partially attributed to similarities between training and test sets. For example, when using the commonly utilized time split approach on the PDBBind set,^17^ the test set contains identical and very similar proteins and ligands, which heavily favors such data-driven methods. These issues as well as ways to deal with them have been discussed at length.^18–21^ Additionally, diffusion based ligand placement methods suffer from some unusual issues, such as alteration of the ligand (e.g., inversion of stereocenters, change of bond orders), which is uncommon with physics inspired methods such as docking. Some of these issues were highlighted by Buttenschoen and co-workers.^22^

Recently, cofolding methods achieved success in a prospective challenge: the pose prediction component of the ASAP-Polaris-OpenADMET antiviral competition, hereafter mentioned as “the Polaris competition”.^23^ The competition involved the prediction of the structure of ca. 200 protein–ligand complexes of inhibitors bound to the SARS-CoV-2 and MERS-CoV Main Protease (MPro). As these true poses were only made available after the challenge concluded, this can be considered an honest benchmark challenge to compare pose prediction methods. In this challenge, cofolding methods outperformed all other methods, particularly traditional pose prediction methods such as FRED,^24^ GLIDE^25^ and Vina,^26^ confirming the reported good performance of cofolding methods cannot solely be attributed to data leakage and similarity. Another success for cofolding methods was reported in the 16th Critical Assessment of Structure Prediction (CASP16) challenge where the AlphaFold3 baseline outperformed all submissions in the protein–ligand pose prediction component of the challenge.^27^

The reported success of the data-driven methods can be partially attributed to the fact a wealth of data exists for the SARS-CoV-2 Main Protease, which is a widely targeted protein,^28^ and which is the target of Nirmatrelvir, Pfizer’s FDA-approved SARS-CoV-2 MPro inhibitor^29^ clinically used to treat COVID-19 infections. For MERS-CoV a much smaller amount of protein–ligand structures are available, although the structure is relatively similar to that of SARS-CoV-2 MPro. The abundance of available protein–ligand crystal structures for SARS-CoV-2 MPro meant that pose information on known ligands could be used as a sort of template or reference to predict the pose of new ligands, with the assumption common parts of different binders will more or less bind in the same location. This same principle is also applied in template-based docking, which is available in FRED, GLIDE and other docking approaches.^30–32^

To further investigate and isolate the power of the template effect, we designed a baseline method which is based completely on ligand alignment. Unlike template-based docking methods or the aforementioned complex data-driven approaches our procedure is based on first finding the best template in a reference ligand set using Maximal Common Substructure (MCS) algorithms, then generating conformations based on the template, using constrained embedding, and then finally ranking conformation by 3D alignment with the template. As such this approach is almost fully ligand-based and only requires the target protein sequence to search a suitable template ligand set for the 3D alignment. The input of the workflow (Figure 1B and 1 **C**) is a set of ligands, for which the pose is to be generated. Given a set of reference protein–ligand complexes (e.g., PDBBind), which are retrieved by looking for similar proteins of an input apo protein (Figure 1A), aligned reference ligands are obtained by alignment and superposition of the reference complexes onto the input protein in 3D space. The aligned reference ligands are then used as input for the TEMPL core pipeline (Figure 1B and 1 **C**).

**Figure 1:**
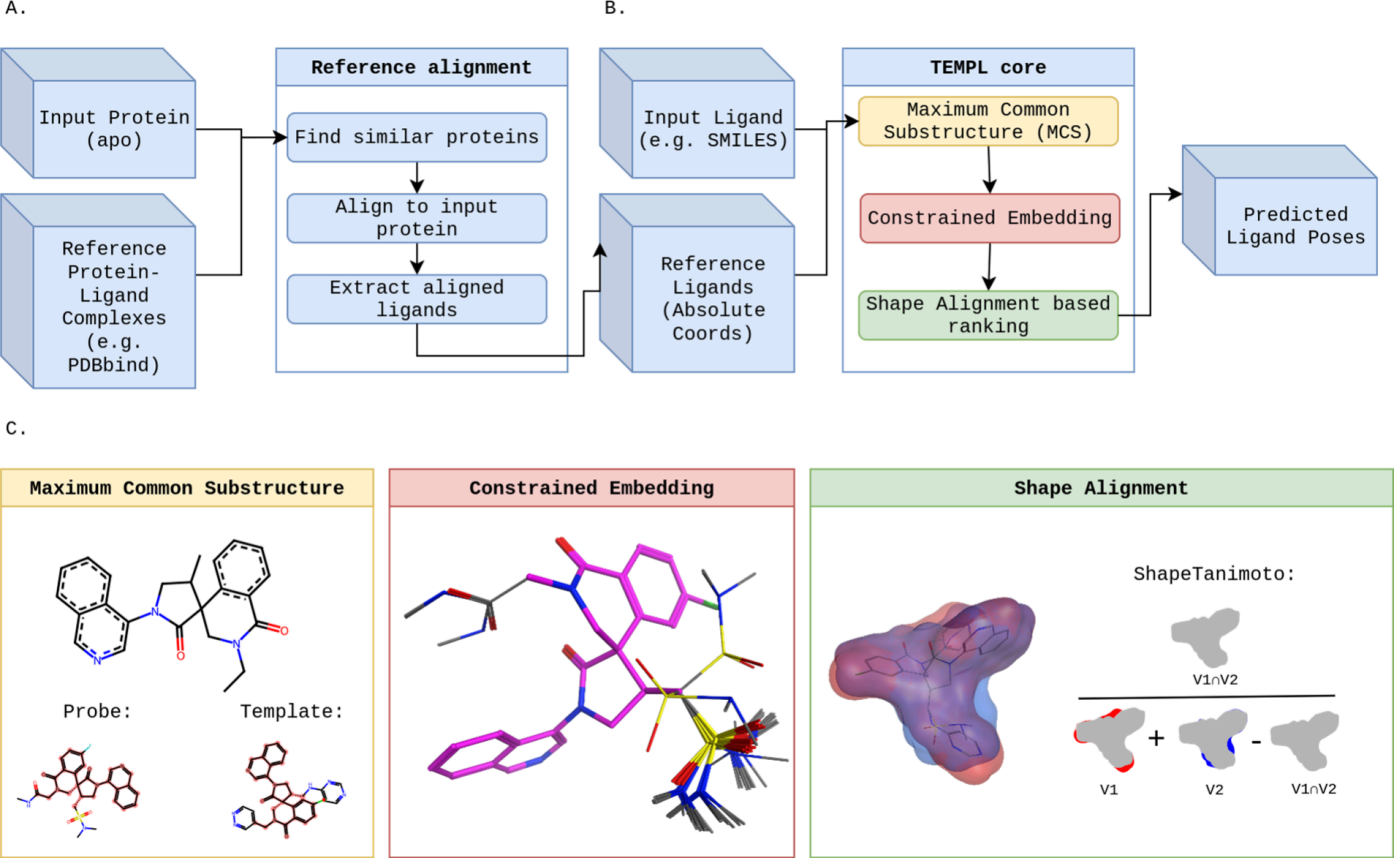
Workflow of TEMPL. (A) Alignment of the input protein to a set of reference protein–ligand complexes can be used to generate the reference ligands to be used as templates. (B) Template-based, ligand-only strategy for placement prediction. (C) Detailed view of every TEMPL core submodule.

## Methods

The core methods of TEMPL are all internal functions of the RDKit,^33^ a cheminformatics library. The procedure used in TEMPL is based on detection of the Maximal Common Substructure (MCS) between the input ligands (with aligned absolute coordinates) and a set of reference ligands. For each input ligand, the best MCS match is used for constrained 3D embedding, generating conformers where the matching atoms from the MCS are locked in the reference coordinates. Then, the generated conformers are ranked using shape or feature alignment to the reference molecule. These correspond to the three blocks in Figure 1B and are described below.

### Maximal Common Substructure

MCS detection was performed using the RascalMCES^34^ algorithm. This method was preferred over the standard MCS method in RDKit (rdFMCS^35^) because of its higher speed, which is an important bottleneck when there is a high amount of probe and reference ligands. This method is different from rdFMCS and leverages matching bonds/edges, and as such is actually based on maximum common edge substructure.

### Constrained Embedding

Constrained embedding was performed using the ETKDGv3 method,^36^ which is a commonly used method for conformer generation,^37^ based on knowledge-enhanced distance geometry. These generated conformers can optionally be optimized using the MMFF94s^38^ or UFF^39^ (in case of atom types that do not have parameters in MMFF94s) force field, although this undoes the strict coordinate constraints from the preceding step.

### Shape Alignment

Finally, ranking of the conformers resulting from constrained embedding was done using RDKit’s Align3D method, which was developed by the PubChem team.^40^ This recently added method is based on the Gaussian volume approximation of molecular shape which allows fast calculation of overlap.^41^ This overlap is quantified using ShapeTanimoto and ColorTanimoto, which corresponds to the intersection of the (colored or not) volumes divided by the union of the volumes. Color here means the gaussians are assigned feature labels, from 6 feature types: hydrogen bond acceptor, hydrogen bond donor, anionic, cationic, hydrophobic and ring features. Another ranking method is ComboTanimoto, which is simply the average between ShapeTanimoto and ColorTanimoto.

### Data Sources

Data related to the Polaris competition was obtained using the Polaris Python tool’s data loader. The competition organizers provided reference complexes for the SARS-CoV-2 and MERS-CoV MPro. These were used as references to align all provided experimental SARS-CoV-2 structures to, using the Biotite Python package.^42^ Only a single reference MERS-CoV complex was provided, but a selection of 17 were extracted from the literature. SARS-CoV-2 MPro ligands were then aligned to the reference MERS-CoV MPro complex to augment the data, as these ligands are chemically much closer to the test set molecules, which both for MERS-CoV and SARS-CoV-2 MPro mostly belong to the same series of isoquinoline-based inhibitors. Finally, the superposed ligands were inspected and anything far from the pocket (e.g., bound to the wrong protein chain) was manually removed. This then led to a set of absolute ligand coordinates which could be used as inputs in the ligand-based core TEMPL pipeline.

For the PDBBind experiments, significant data cleaning was performed on the PDBBind^17^ (v2020) data set. To ensure correct parsing of molecules, which are provided as SDF files, the corresponding ligand SMILES were retrieved from the work of Li and co-workers^43^ and used to reconstruct and correct the atom connectivity, which is essential for correct detection of MCS. We rejected all molecules that could not be parsed by RDKit, and additionally, we filter out oligopeptides with more than 8 residues and oligosaccharides with more than 3 sugars, as our method is intended for small molecule pose prediction and very large molecules are not compatible with MCS algorithms. In the end, this resulted in a training set size of 15119 complexes, a test set size of 340 complexes and a validation set size of 883 complexes.

PoseBusters data was used as provided.^22^

The methods used for reference alignment are shown in Figure 1A and are described below.

### Finding Similar Proteins

The sequences of PDBBind protein chains were embedded using ESM2 ^44^ (specifically the esm2_t33_650M_UR50D model), averaging the per residue embeddings into a single 1280 float embedding vector for each input protein sequence. The distance between different sequences were quantified using cosine distance, with the best 100 template proteins retained. To increase portability of our method, we have also made the used embedding available via a publicly available repository to spare the user the effort of having to recalculate these for all proteins in the PDBBind set.

### Protein Superposition

For protein backbone superposition, we applied the “superimpose homologs” method from Biotite. When the final Cα RMSD was above a specified threshold (default of 10 Ångström), the protein was discarded due to low agreement between the structures (in case no protein at all is found, as a fallback, the distance threshold is relaxed with 5 Å steps in order to have at least one protein template).

### Extract Aligned Ligands

The transform (i.e., rotation and translation) used during protein superposition is applied on the corresponding ligand, transforming the coordinates of the ligand in the data set. These absolute coordinates are stored in the SDF file used as input in the TEMPL core pipeline.

### Pose Assessment

For calculation of ligand pose Root Mean Square Deviations (RMSDs), the sPyRMSD Python package^45^ was used. This method is symmetry-corrected and is also independent of hydrogens and bond orders, which enables some tautomer and protomer related nonidentity issues to be averted. This is the same RMSD implementation which was used for the Polaris competition leaderboard. The equation for RMSD is given below (eq 1), with A and B the N by 3 matrices of atomic coordinates of two conformers A and B with matched heavy atoms.

$$\mathrm{RMSD}=\sqrt{\frac{1}{N}\sum_{1}^{N}\sum_{0}^{2}(A_{ij}-B_{ij}{)}^{2}}$$ 1

Generally, an RMSD of 2 Ångström and below has been considered a docking success although this threshold is considered crude and a truly successful docking prediction will also involve other factors such as physical soundness, retrieval of molecular interactions and realistic torsion angles.^22^

As a second metric for assessing ligand pose predictions, we apply Local Distance Difference Test Protein–Ligand Interactions (lDDT-PLI), a variant of lDDT^46^ which looks at the conservation of protein–ligand contacts, and which is commonly used to assess the quality of predicted protein–ligand structures.^47^ We used the docker image provided by OpenStructure,^48^ using standard settings and we considered any failure or error as a score of 0.000.

## Results

### Polaris Competition Pose Prediction

A prototype version of our method was applied in the pose prediction component of the Polaris competition. This challenge involved predicting the pose of a series of ca. 200 closely related ligands to the SARS-CoV-2 and MERS-CoV MPro. A training set of about 800 complexes to SARS-CoV-2 Main protease was made available as a part of the challenge. The initial prototype of our method achieved a success rate of 51% (MERS-CoV + SARS-CoV-2 < 2 Å) on the intermediary leaderboard. On the final leaderboard, the performance of the baseline deteriorated to 34%,^23^ mainly due to using a smaller template ligand set for the MERS-CoV Main protease and not using SARS-CoV-2 MPro data. Below, we report the results for the finalized version of TEMPL, which was developed after the Polaris competition ended (incorporating a web server, code cleanup, an ablation study, and data cleanup). We performed an ablation study, of which the results are summarized in Table 1. The final achieved performance using the optimized settings is 75.4% (MERS-CoV + SARS-CoV-2 < 2 Å) and lDDT-PLI of 0.838.

**Table 1:** Ablation Study (Metrics Reported for Test Data)^a^

experiment	settings	MERS-CoV < 2 Å	SARS-CoV-2 < 2 Å	MERS-CoV+SARS-CoV-2 < 2 Å	lDDT-PLI (mean + - st.dev.)
(A) default	MCS + 200 conformations + ComboTanimoto	67.0	74.5	70.8	0.838 ± 0.150
(B) different ligand templates	MERS PDB reference only (instead of transposed SARS-CoV-2)	16.5	74.5	45.6	0.720 ± 0.230
(C) different 3D alignment score	ShapeTanimoto	62.9	68.4	65.6	0.721 ± 0.267
	ColorTanimoto	59.8	69.4	64.6	0.785 ± 0.186
(D) no constrained embedding	unconstrained embedding + ComboTanimoto	66.0	72.4	69.2	0.782 ± 0.200
	unconstrained embedding + ShapeTanimoto	63.9	69.4	66.7	0.775 ± 0.206
	unconstrained embedding + ColorTanimoto	58.8	68.4	63.6	0.775 ± 0.208
(E) no realignment	no realignment	64.9	69.4	67.2	0.725 ± 0.190
(F) final force field minimization	MMFF94 optimization	60.8	68.4	64.6	0.750 ± 0.194
(G) reduced conformations	100 conformations	62.9	74.5	68.7	0.777 ± 0.205
	50 conformations	60.8	69.4	65.1	0.777 ± 0.199
	20 conformations	53.6	63.3	58.5	0.759 ± 0.199
	10 conformations	42.3	63.3	52.8	0.753 ± 0.187
	5 conformations	28.9	52.0	40.5	0.700 ± 0.228
	1 conformation	15.5	19.4	17.4	0.557 ± 0.266

a (A) Default settings. (B) Different ligand templates (literature MERS-CoV MPro complexes only, instead of transposed SARS-CoV-2 MPro ones). (C) Different 3D alignment scoring function. (D) No constrained 3D embedding (i.e., no locking of coordinates based on MCS). (E) No realignment (keep coordinates fixed during 3D alignment, so direct output of 3D embedding reranked with alignment scores only). (F) To minimize the final structure, a force field (MMFF94s) was used. (G) Reduced amount of ligand conformations used in the pipeline.

### PDBBind Pose Prediction

To compare our baseline head to head with recent deep learning pose prediction methods, we employed the PDBBind data set, using commonly used splits, in particular the time split used by Strk and co-workers.^8,9^ The PDBBind data set is a curated subset of the Protein Data Bank (PDB) with protein–ligand complexes, which consists of 18,902 unique structures before postprocessing. This split was later criticized for test-train similarities, both on the ligand and protein level, making the pose prediction task easier than anticipated and possibly inflating the performance of data driven pose prediction methods. Because our baseline is designed to capture such effects, we anticipated this is one place where our baseline method should show some degree of successful pose predictions due to near-neighbor behavior.

Using these provided splits, which is based on a cutoff at a given date (first of January 2019), and using the PDBBind train set complexes as a reference, TEMPL achieves an RMSD < 2 Å success rate on the test set of 22.1%. This can be compared with the reported success rates of GLIDE at 21.8%, EquiBind at 5.5% and DiffDock at 38.2%.^9^ Note that reported GLIDE numbers are relatively low because the reported task is blind docking, so without the binding site specified, which is typically specified for traditional docking algorithms such as GLIDE. In Figure 2, the success rate dependency on training set similarity is shown both for ligand and protein similarity. As expected, particularly in the situation of high ligand similarity combined with high protein similarity, high success rates (67.3%) are achieved.

**Figure 2:**
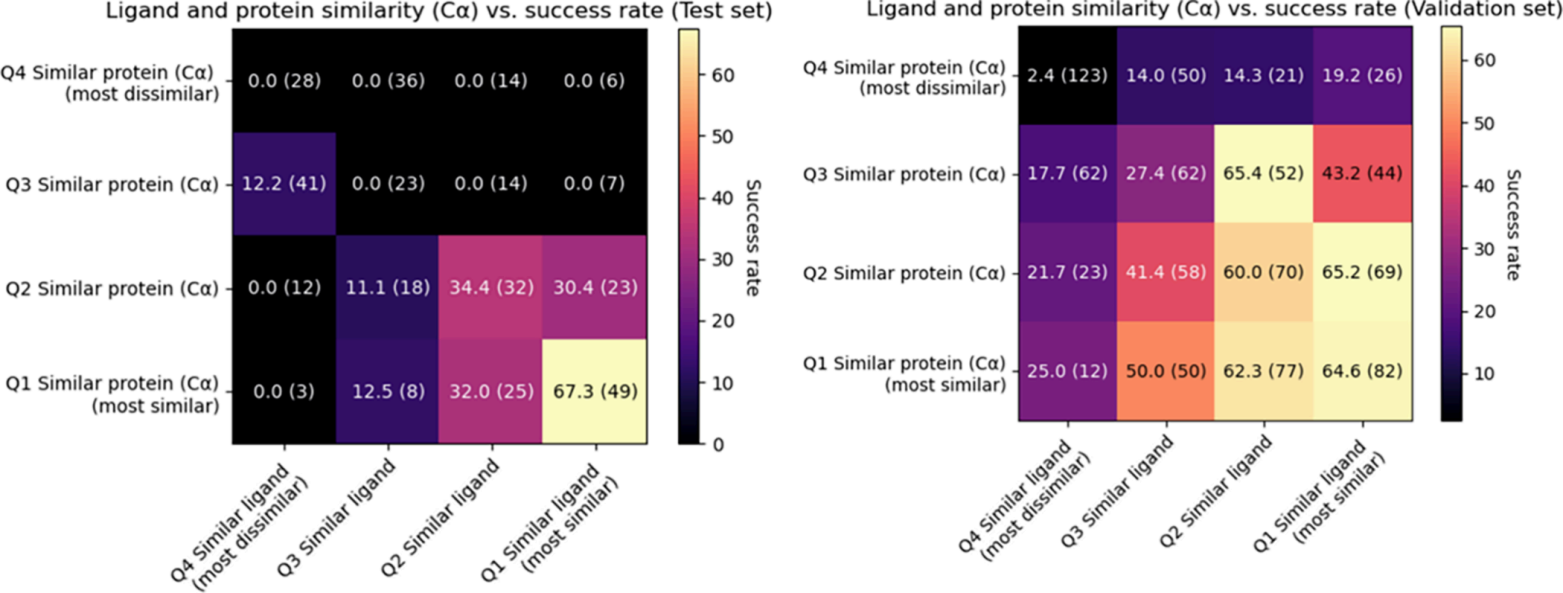
RMSD < 2 Å success rate versus protein and molecular similarity for the test set and for the validation set. Protein similarity is estimated by RMSD of Cα between input protein and closest found template protein after homologous superimposition with Biotite, molecular similarity using the Tanimoto similarity of ECFP4 (2048 bits). Binning is done by quartile (Q1–Q4). Numbers between brackets are the size of the bin.

The validation set achieved a higher pose recovery rate of 41.3%, this is a consequence of the fact the validation set is a random split of the pre-2019 PDBBind train set and as such has higher average similarity to the training set. We also performed a Leave-One-Out (LOO) experiment on the train set itself (where all train set templates except the one for the current pose prediction are used), achieving a similar pose recovery rate of 42.3%.

From the point of view of lDDT-PLI a similar tendency is present: the leave-one-out train task achieves an lDDT-PLI of 0.581 ± 0.330, the test set is worse at 0.346 ± 0.351 and the validation set 0.577 ± 0.329.

### PoseBusters Pose Prediction and Pose Evaluation

One of the first serious challenges to the reported good performance of data-driven pose placement methods was made by the PoseBusters team, which showed these methods suffer from serious problems including physically improbable conformations and steric clashes.^22^ The PoseBusters authors also provided a more challenging protein–ligand complex data set which was later used as a benchmark set for cofolding methods such as AlphaFold3. Using the same workflow, TEMPL achieved *a* < 2 Å success rate of 8.9% (<5 Å success rate of 17.6%), which outperformed EquiBind (2.0%), but was outperformed by all other DL methods (reported success rates were TankBind 16%, DeepDock 20%, Uni-Mol 22%, DiffDock 38%) and compared to traditional methods such as AutoDock Vina (60%). The lDDT-PLI is similarly low, obtaining a mean value of 0.189 ± 0.305, corresponding to a low success rate.

In Figure 3, the success rate dependency on training set similarity is shown both for ligand and protein similarity. Similarly to the PDBBind case, in the situation of high ligand similarity combined with high protein similarity, the highest success rates (53.3%) are achieved.

**Figure 3:**
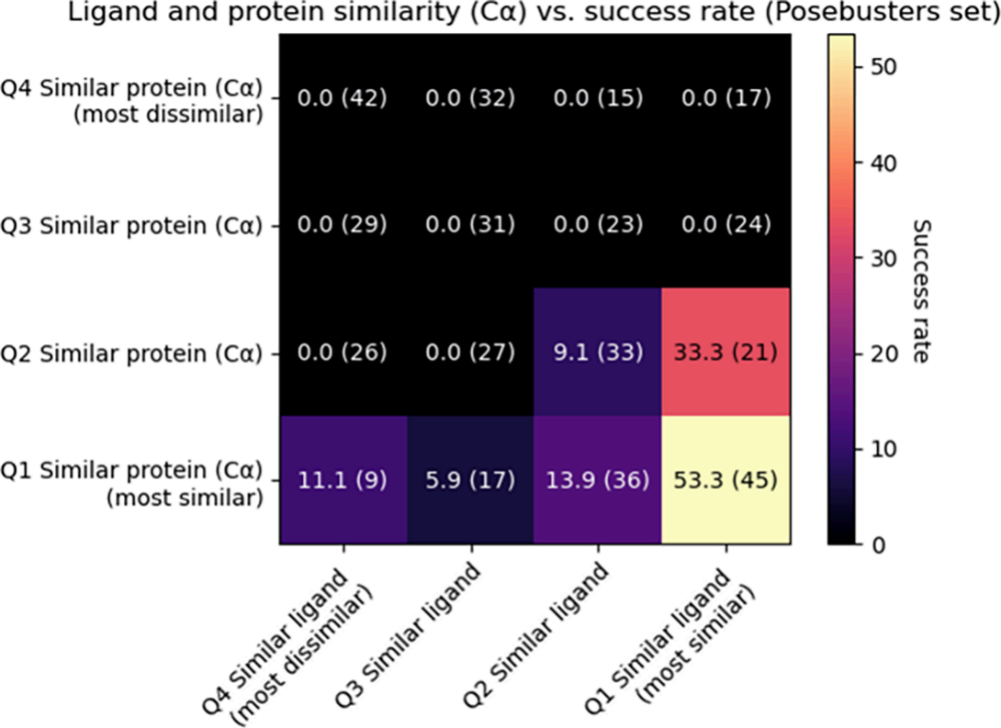
Performance on the PoseBusters task is strongly dependent on similarity of ligands and proteins to the reference set. Protein similarity is estimated by RMSD of Cα between input protein and closest found template protein after homologous superimposition with Biotite, molecular similarity using the Tanimoto similarity of ECFP4 (2048 bits). Binning is done by quartile. Numbers between brackets are the size of the bin.

Additionally, we used PoseBusters to estimate the PoseBusters validity of poses obtained using TEMPL. As TEMPL is based on MCS alignment and is strictly ligand-based, there is no awareness at all of possible steric clashes, and the aim is solely to obtain poses that perform well on the RMSD based metric. As expected, 66.7% of the obtained poses within the <2 Å threshold were PoseBusters-invalid. The invalidity rate is comparable with the invalidity rates reported for DL method DiffDock (68%), and better than the other DL methods (EquiBind 100%; TankBind 79%; DeepDock 74%; Uni-Mol 91%) but significantly higher than the low invalidity reported for AF3 and traditional docking methods such as AutoDock Vina (<5%). The main cause of invalidity was failure on the “Minimum protein–ligand distance” task, meaning there are unrealistically close atom distances between the ligand and the protein.

### Software Package

Our method is offered as a Python-based command-line tool, and additionally, the tool is made available as a web application, with the intention to facilitate its usage as a baseline method to compare against other data-driven methods. The web application is based on Streamlit and makes it possible for users to try out the TEMPL pipeline without commitment, local installation or familiarity with command-line software. The web application can be run locally, but we have made an instance of it available via https://templ.dyn.cloud.e-infra.cz. Instruction for a basic usage task for both the command line tool and the web app are given in the Supporting Information. A detailed flowchart of the workflow including all fallbacks is available in the code repository.

## Discussion

The pose prediction experiments described in the results section of this paper confirm the initial observation from our submission to the Polaris competition. When similar protein–ligand complexes are available, pose prediction based on ligand similarity achieves high success rates. As described elsewhere^18–20^ the same effect leads to overestimation of the strength of data-driven pose prediction methods.

The Polaris competition itself corresponded to a best-case scenario for similarity-based methods: a large amount of protein–ligand complex structures were available for SARS-CoV-2 Main Protease and the ligands in the test set mostly belonged to one series of similar compounds that all contain a decorated isoquinoline. In this case, our method performed well and outperformed traditional and physics-based methods, including AutoDock Vina with template placement correction. On the other hand, fine-tuned deep learning methods performed significantly better, occupying the entire top 3 in the final leaderboard.^23^

The ∼ 20% increase in final reported performance of 70.8% pose recall for TEMPL in the Polaris competition versus the intermediary leaderboard performance of 51% can be attributed to enhanced data cleanup, polishing of the prototype, and the capacity of running the ablation study on unblinded test data.

The ablation study (Table 1) showed all 3 components, MCS, constrained embedding and alignment based placement ranking are necessary for good performance (Table 1A). It was also shown that force field optimization of poses decreased the success rate (Table 1F). It is known ETKDGv3 generated conformers create very high quality ensembles of conformations without further optimization,^37^ but based on previously known results,^37^ force field optimization is not expected to make the performance worse, or to lower conformation diversity. However, there is an obvious reason for the degraded performance in our case: force field optimization loosens coordinate constraints of formerly locked atoms.

Furthermore, the ablation study showed that 3D alignment on its own (i.e., without any MCS or template) is able to capture many correct poses too (Table 1D), although it underperforms relative to our optimized baseline (Table 1A). When 3D alignment was used for ranking only (and not for optimization of the coordinates), there was a slight degradation in performance (Table 1E). It was found 200 conformers per input molecule are necessary (Table 1G), in line with the observations by McNutt and co-workers.^37^ The choice of reference molecules is very important: realigned SARS-CoV-2 MPro data performed much better than MERS-CoV MPro data alone (Table 1B). This confirmed our intuition that similar protein templates with similar ligands give more meaningful ligand templates than identical protein templates with less similar ligands. For 3D alignment, ComboTanimoto outperforms both ShapeTanimoto and ColorTanimoto (Table 1C), suggesting feature distribution and molecular shape contain complementary information that is useful for successful alignment.

Applying this method on widely used pose prediction benchmarks, PDBBind and PoseBusters, confirmed a strong dependency on similarity and, in the case of the PDBBind benchmark, achieved moderate performance, comparable with reported numbers for traditional docking methods. On novel proteins and/or using novel ligands, the performance completely collapses, confirming this approach is, unlike traditional docking methods,^19^ not able to extrapolate meaningfully at all (Figure 2). This was particularly clear on the more novel PoseBusters benchmark set, where there is essentially no pose recovery outside of the known region (Figure 3).

Many of the output poses on the PoseBusters task did not pass the validity checks, although surprisingly, more than 33% were valid, which is a higher validity rate than all the other deep learning based methods in this benchmark. This is interesting, because all information about possible steric clashes or protein contacts is only indirectly provided by the shape of the reference ligands. Nonetheless, this is apparently enough to prevent steric clashes in many cases. Because ligand conformations in our method originate from ETKDGv3, only a limited amount of physically unrealistic conformers occur.

TEMPL is a baseline method and is expected to underperform in challenging tasks. As discussed above, this is seen in the PoseBusters protein–ligand benchmark, where we observed a low recovery rate of correct poses. Comparably with what is reported for deep learning based methods in the PoseBusters pose prediction task, we observe a distinct relation between pose recovery success and the similarity of protein sequences to the training set. Notably, modern cofolding methods such as AlphaFold3 perform well on this task. We propose our method as a challenging data-driven baseline that should be considered the minimum acceptable performance for any advanced data-driven method, including not only pose recall but also PoseBusters validity.

## Conclusions

We have constructed a baseline method which leverages knowledge from known protein–ligand complexes to predict the poses of new ligands to new proteins. We show this baseline achieves relatively strong performance at pose prediction, sometimes outperforming widely used traditional pose prediction methods such as AutoDock Vina. This is surprising, because the method is ligand-based without any consideration of protein–ligand binding such as molecular interactions or steric factors. Although our baseline was not among the highest ranked in the final Polaris competition leaderboard, the final optimized baseline outperforms many advanced pose prediction techniques and matches the performance of the best physics-based methods. We propose our baseline to be used as a realistic minimal baseline that needs to be outperformed by newly proposed pose prediction methods, particularly under conditions where highly similar protein or ligand information can be leveraged. Our method is available both as open-source code as well as through a web app, accessible at https://templ.dyn.cloud.e-infra.cz/.

## Supplementary Material



## Data Availability

The source code of TEMPL is freely available via github.com/fulopjoz/templ-pipeline, and is released under the permissive MIT license. This also includes the code for a streamlit application, which can be run locally or accessed via https://templ.dyn.cloud.e-infra.cz/. Data is deposited to Zenodo, including a snapshot of the code at the time of submission and precalculated ESM embeddings, and including outputs of benchmarks. Benchmark output data are available via https://zenodo.org/records/16875932.

## Notes

Code examples for basic usage; basic usage of the web server (Figure S1); benchmark output data summarized in Tables S1 and S2; similarity quartile heatmaps using ESM2 cosine distance as the similarity metric (Figure S2); relation between RMSD and lDDT-PLI (Figure S3); lDDT-PLI per protein target class (Figure S4) (PDF)
