# Supplementary material for: TEMPL: A Template-Based Protein–Ligand Pose Prediction Baseline
Source: J Chem Inf Model. 2025 Oct 14;65(20):11149–57. doi: 10.1021/acs.jcim.5c01985 (PMC12570141; doi:10.1021/acs.jcim.5c01985)
Supplement: Supplementary file 1 [file ci5c01985_si_001.pdf]

TEMPL: A template-based protein ligand pose prediction baseline

Supporting Information

Jozef Fülöp[1], Martin Šícho[1], Wim Dehaen[1][2]*
[1] CZ-OPENSCREEN, Department of Informatics and Chemistry, Faculty of Chemical Technology, University of Chemistry and Technology Prague, Technická 5, 16 628 Prague 6, Czech Republic;
[2] Department of Organic Chemistry, Faculty of Chemical Technology, University of Chemistry and Technology Prague, Technická 5, 16 628 Prague 6, Czech Republic;
*Corresponding author. E-mail: dehaenw@vscht.cz

Code examples for basic usage

Simple pose prediction:

```
 templ run --protein-file data/example/1iky_protein.pdb --ligand-smiles
"CCO"
```

Using PDB ID instead of file:

```
  templ run --protein-pdb-id 1iky --ligand-smiles "CCO"
```

Using SDF file for ligand:

```
  templ run --protein-file data/example/1iky_protein.pdb --ligand-file
data/example/1iky_ligand.sdf
```

With optimization enabled:

```
  templ run --protein-file data/example/1iky_protein.pdb
--ligand-smiles "CCO" --enable-optimization
```

Web app basic usage

Shown in the screenshot below: The user provides ligand information as SDF or SMILES in **1**, provides either the protein of interest (as pdb code or pdb file) or provides a set of templates for ligand-based alignment as sdf in **2**. This is everything needed to submit the job for prediction in

3. After the calculation is finished, a results tab appears in 4. This will show the matched template, closest ligand and MCS. Results can be downloaded via the button on the bottom in 5.

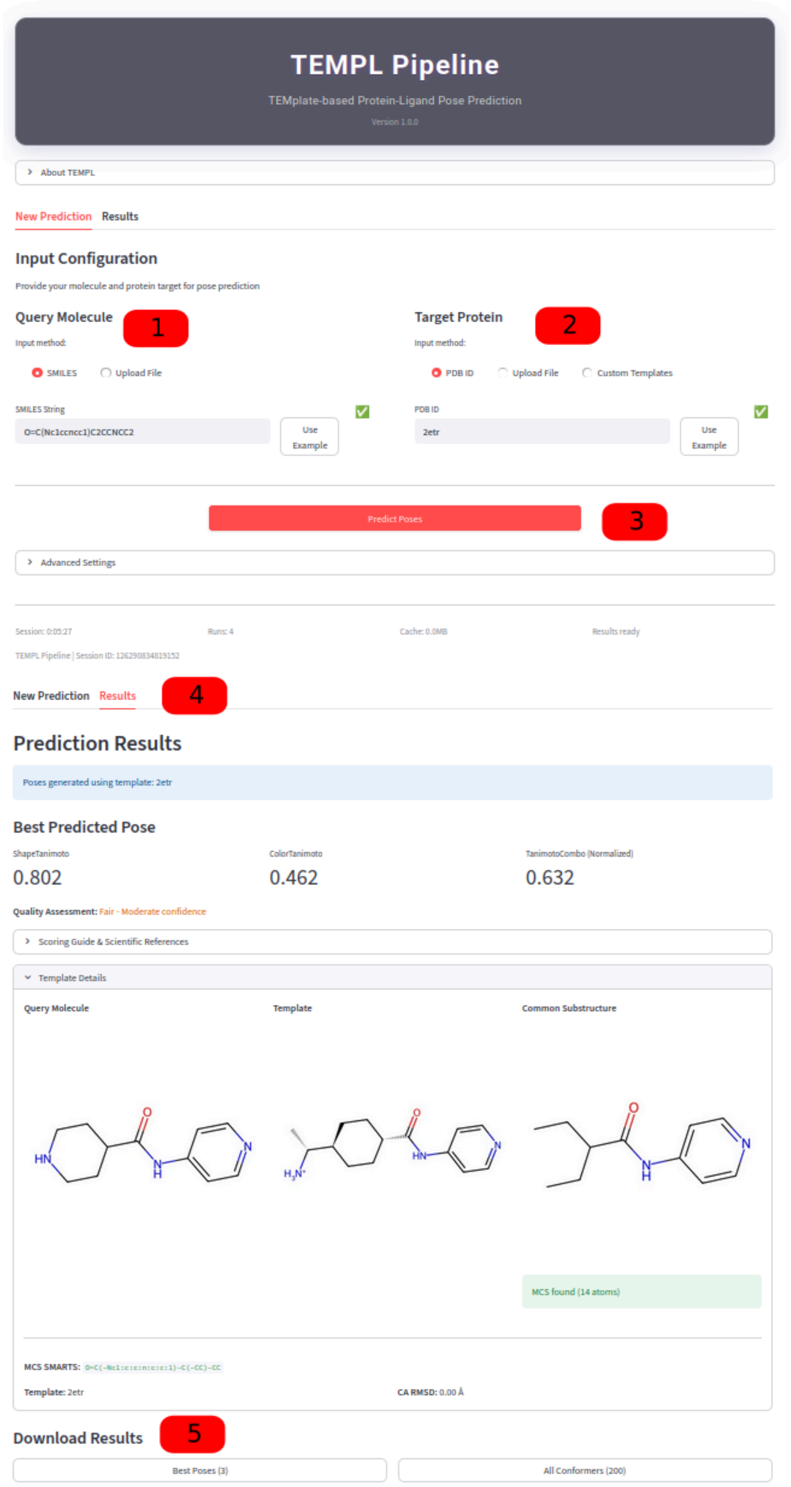

Figure S1: screenshot of the TEMPL web interface, accessible via https://templ.dyn.cloud.e-infra.cz/

Benchmark full outputs

Polaris

Set	Templates	< 2 Å (%)	< 5 Å (%)
SARS Train	SARS	67.0	89..2
SARS Test	SARS	74.5	92.9
MERS Train	MERS	23.5	76.5
MERS Test	MERS	16.5	47.4
MERS Train	MERS+realigned SARS	35.3	88.2
MERS Test	MERS+realigned SARS	67.0	99.0

Table S1: full benchmark results for the Polaris task

PDBBind

Set	Alignment	< 2 Å (%)	< 5 Å (%)
Train (LOO)	Color	38.9	67.6
Train (LOO)	Combo	42.3	67.9
Train (LOO)	Shape	40.9	64.7
Test	Color	18.2	38.8
Test	Combo	22.1	37.9
Test	Shape	19.7	37.1
Validation	Color	37.6	66.5
Validation	Combo	41.3	67.2
Validation	Shape	38.9	63.5

Table S2: Full benchmark result for the PDBBind task. The template source is Train PDBBind data in all case, in case of the Train results, the reported result are Leave One Out (LOO))

Posebusters Ligand-Protein Similarity Heatmaps

using ESM2-cosine distance as a similarity function.

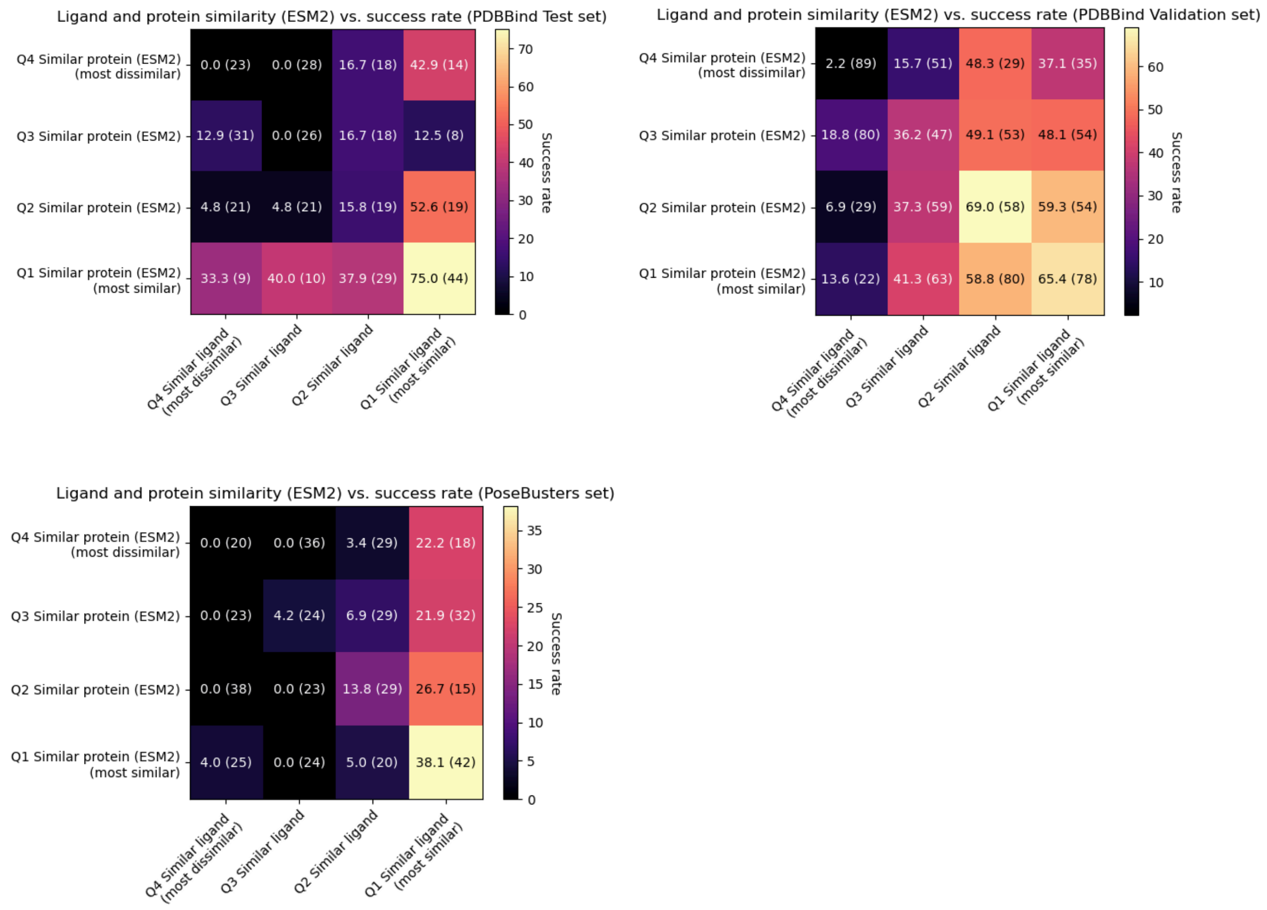

Figure S2: alternative visualization of protein distance using ESM2 cosine distance.

lDDT-PLI versus RMSD

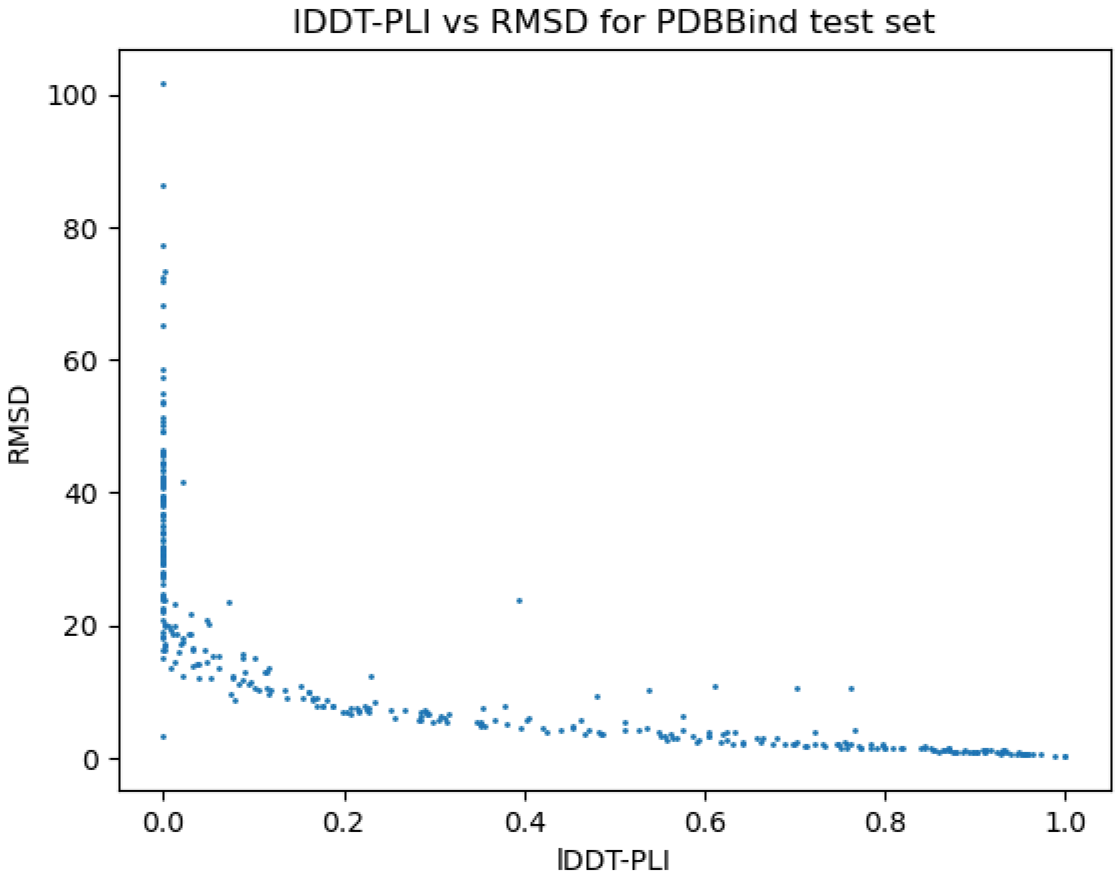

Figure S3: lDDT-PLI mostly closely tracks RMSD, with lDDT-PLI close to 1 corresponding to low RMSDs and lDDT-PLI close to 0 corresponding to high RMSDs.

lDDT-PLI versus protein category

Protein categories were annotated using the Interpro API based on the uniprot ID. 8 categories were used: Nuclear receptors, GPCRs, Kinases, Hydrolases, Proteases,Transporters, Ion Channels, and anything that does not fit into the preceding categories. The data shown is for the leave-one-out PDBBind set (which had the largest and most diverse amount of proteins)

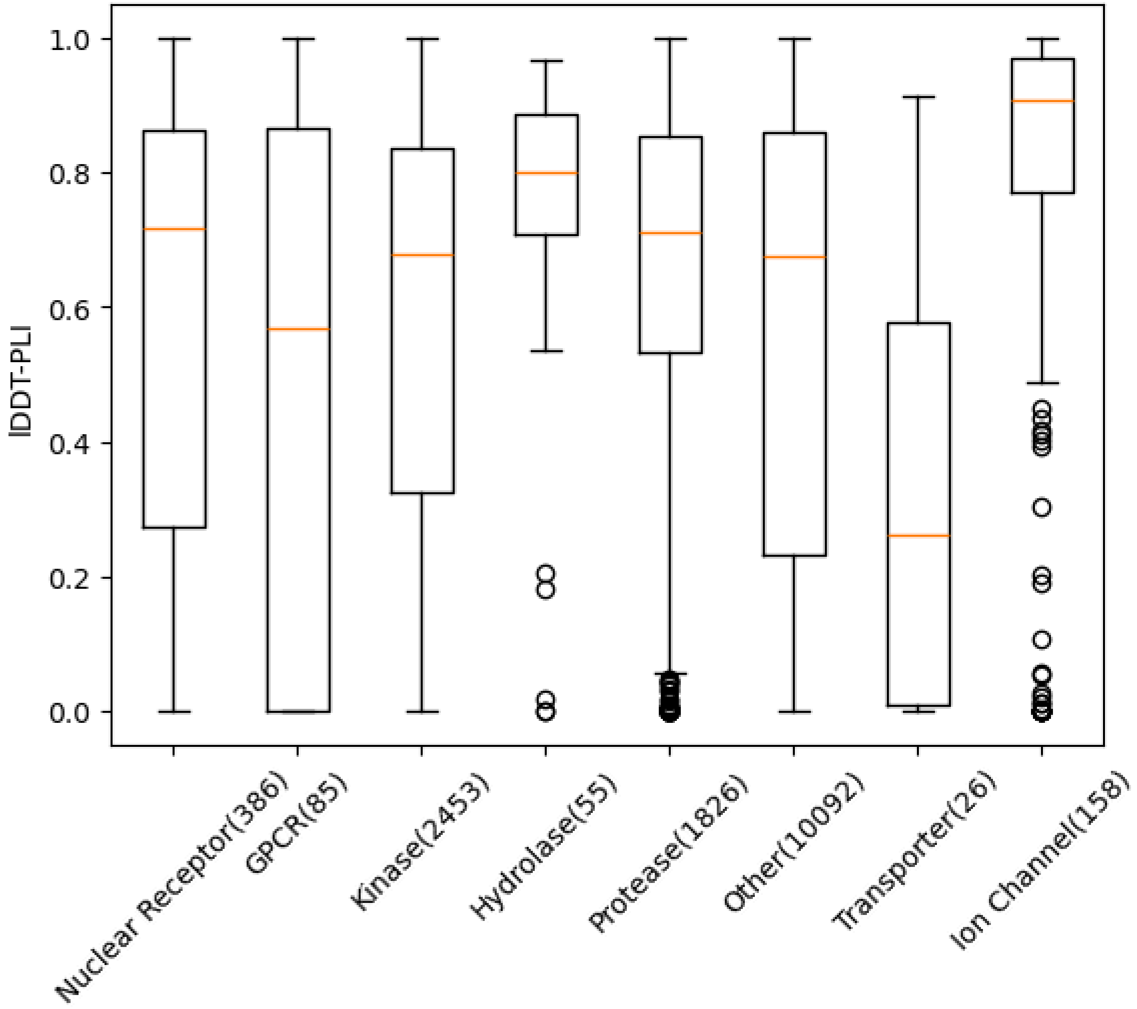

Figure S4: Box plots showing lDDT-PLI differences among different protein target groups. The number between parentheses is the amount of proteins in that category.
